# Stretchable Electrospun PVDF-HFP/Co-ZnO Nanofibers as Piezoelectric Nanogenerators

**DOI:** 10.1038/s41598-017-19082-3

**Published:** 2018-01-15

**Authors:** Hemalatha Parangusan, Deepalekshmi Ponnamma, Mariam Al Ali Al-Maadeed

**Affiliations:** 1 Center for Advanced Materials, Qatar University, P O Box 2713, Doha, Qatar; 2 Materials Science & Technology Program (MATS), College of Arts & Sciences, Qatar University, Doha, 2713 Qatar

**Keywords:** Materials for energy and catalysis, Electronic properties and materials

## Abstract

Herein, we investigate the morphology, structure and piezoelectric performances of neat polyvinylidene fluoride hexafluoropropylene (PVDF-HFP) and PVDF-HFP/Co-ZnO nanofibers, fabricated by electrospinning. An increase in the amount of crystalline β-phase of PVDF-HFP has been observed with the increase in Co-doped ZnO nanofiller concentration in the PVDF-HFP matrix. The dielectric constants of the neat PVDF-HFP and PVDF-HFP/2 wt.% Co-ZnO nanofibers are derived as 8 and 38 respectively. The flexible nanogenerator manipulated from the polymer nanocomposite (PVDF-HFP/Co-ZnO) exhibits an output voltage as high as 2.8 V compared with the neat PVDF-HFP sample (~120 mV). These results indicate that the investigated nanocomposite is appropriate for fabricating various flexible and wearable self-powered electrical devices and systems.

## Introduction

Owing to the escalating worldwide concern over energy calamity and environmental issues, attention has been diverted towards future energy. Recently, rechargeable power and renewable energy sources have been considered as an attractive alternative for environmental problems^1^. Among them, mechanical energy is one of the most abundant energy sources, and easily convertible to other useful energy forms. Piezoelectric materials can convert the mechanical energy produced by various activities into electrical energy. The mostly employed piezoelectric materials include barium titanate and lead zirconate titanate, which exhibit superior efficiency for energy conversion and high piezoelectric constants. However, these materials show disadvantages such as cost intensive nature, toxicity, brittleness and they are non- environmentally friendly. In order to overcome these problems, the research community is strongly motivated to identify new piezoelectric materials that are lightweight, flexible, biocompatible and electroactive^2–7^.

Some of the semi crystalline polymers such as polyvinylidene fluoride (PVDF) and its copolymers are well known materials for fabricating piezoelectric devices^8^. These polymers are used in many fields, such as portable electronic devices, flexible pressure sensors, energy harvesting systems, water purifying devices and in gas separation^8–10^. In the neat form, these polymers have poor electrical and mechanical performance and the enhancement of their piezoelectricity is still a challenge^11^. Reports suggest the addition of nanofillers in various dimensions to PVDF and its copolymers like polyvinylidene fluoride hexafluoropropylene (PVDF-HFP) to enhance the piezoelectric, pyroelectric and ferroelectric performances. The piezoelectricity of PVDF-HFP is mainly contributed by polar crystalline phases such as β-phase and γ-phase, rather than the α-phase. Among the various crystalline phases, the electrocactive β- phase imparts the highest dipole moment resulting in high piezoelectricity^12^.

The β- phase content of such polymers can be increased by various techniques, such as stretching, combined stretching and poling of polymer films or fiber formation methods at given temperatures. Among the various techniques, electrospinning is the most effective in producing self- poled piezoelectric nanofibers because of the high stretching forces exerted on electrified solution jets. However, PVDF-HFP films or fibers partially depolarize after mechanical stretching and even electrospinning via thermal motion can bring back the polymer to the stable curled state. Recently, the addition of nanofillers has emerged as an alternative chemical method to increase the β-phase content by introducing specific polymer-filler interactions and stabilize the β-phase nanocrystals^13–15^.

Here we report the fabrication of PVDF-HFP/Co-doped ZnO composite nanofibers by electrospinning method, and their piezoelectric properties. To the best of our knowledge, no prior work regarding the piezoelectric properties of PVDF-HFP/Co-ZnO nanocomposites has been reported till date. To investigate their structural, morphological and dielectric behaviours, the nanofibers were characterized by X-ray diffraction technique (XRD), Fourier transform infrared spectroscopy (FTIR), scanning electron microscope (SEM). The composite containing 2 wt.% of Co-doped ZnO nanoparticles achieved a piezoelectric performance of 2.8 V. The detailed investigation on the mechanism related to the piezoelectric property has also done.

## Experimental details

PVDF-HFP (M_w_ ~ 4,00,000), dimethylformamide (DMF) and acetone were purchased from sigma Aldrich. Zinc acetate dehydrate [Zn(CH_3_ COO)_2_.2H_2_O], polyethylene glycol (PEG), monoethanolamine (C_2_H_7_NO), cobalt chloride were also purchased from Sigma Aldrich.

Co-doped ZnO and undoped ZnO nanoparticles were prepared by hydrothermal method. In this process, zinc acetate and cobalt chloride salts were dissolved in water and stirred for 2 h to obtain a clear and homogeneous solution. To avoid the agglomeration of nanoparticles, PEG was added to the resultant solution which was used as a surfactant and again stirred for another 1 h. The resulting solution was transferred to Teflon capped autoclave and kept at 140 °C for 15 min. After the reaction process, the autoclave was cooled down to room temperature. The obtained precipitate was washed with water and ethanol, and the whole process was repeated for the neat ZnO. Finally, the powders were dried at 80 °C in a hot air oven for 12 h and then annealed in tube furnace at 400 °C for 2 h.

PVDF-HFP solution was prepared by the addition of 2 g PVDF-HFP pellets to a 1:1 mixture of DMF and acetone (15 ml solvent) and stirred for 3 h at 70 °C. The undoped ZnO (1 wt.%) and Co-doped ZnO (0.5, 1 and 2 wt.%) nanopowders were dispersed in the same solvent mixture (5 ml) and then sonicated for 2 h. The dispersed filler solutions were mixed with each of the PVDF-HFP solution and the whole mixture was magnetically stirred overnight to obtain a homogeneous mixture. Finally, those solutions were used for electrospinning.

**Figure 1:**
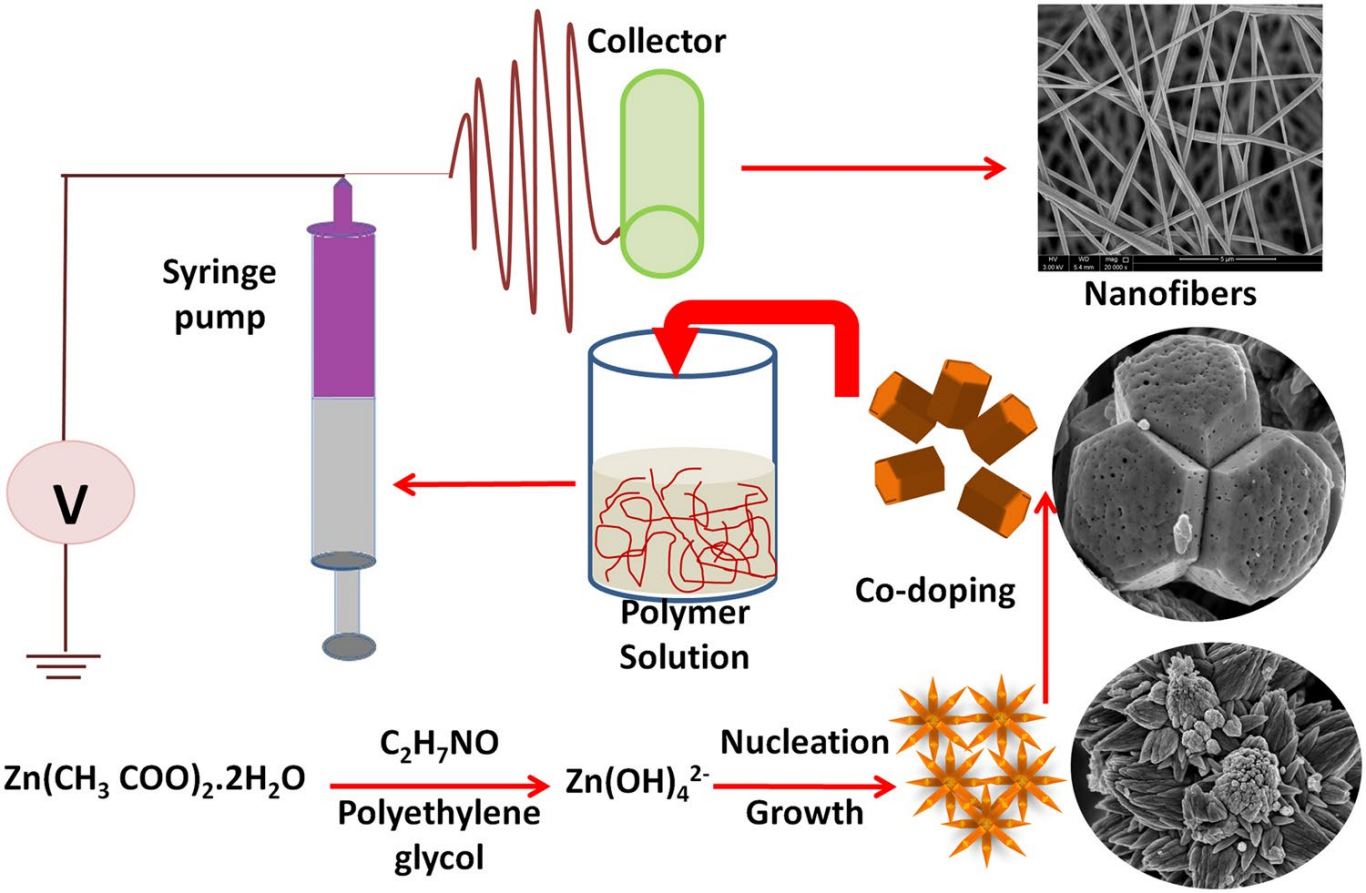
Schematics of the sample preparation and the electrospinning setup.

Figure 1 shows the sample preparation procedure and the electrospinning setup to obtain the composite nanofibers. The PVDF-HFP and its nanocomposite solutions were electrospun at a rate of 1 ml/h and the nanofibers were collected in a rotor, rotating at a speed of 500 rpm. The tip-to collector distance was fixed as 15 cm and the applied voltage was 12 kV after optimization. The electrospun nanofibers were collected on an aluminium foil substrate pasted on the collector.

The morphology and structure of the nanopowders and the polymer composites were investigated by scanning electron microscope (SEM, XL-30E Philips Co., Holland), transmission electron microscope (FEI TECNAI G^2^ TEM), XRD diffractometer (Mini Flex 2, Rigaku equipped with Nickel filtered CuKα radiation (λ = 0.1564 nm) operated at 30 V and 15 mA in the 2θ range of 10–30° at a scanning speed of 1.8°/min) and FTIR spectroscope (PerkinElmer Spectrum 400 spectrophotometer in the range 400–4000 cm^−1^ with a resolution of 2 cm^−1^). Dielectric properties were tested by broadband dielectric/impedance spectroscopy-Novocontrol at a frequency range of 10^1^ to 10^7^.

**Figure 2:**
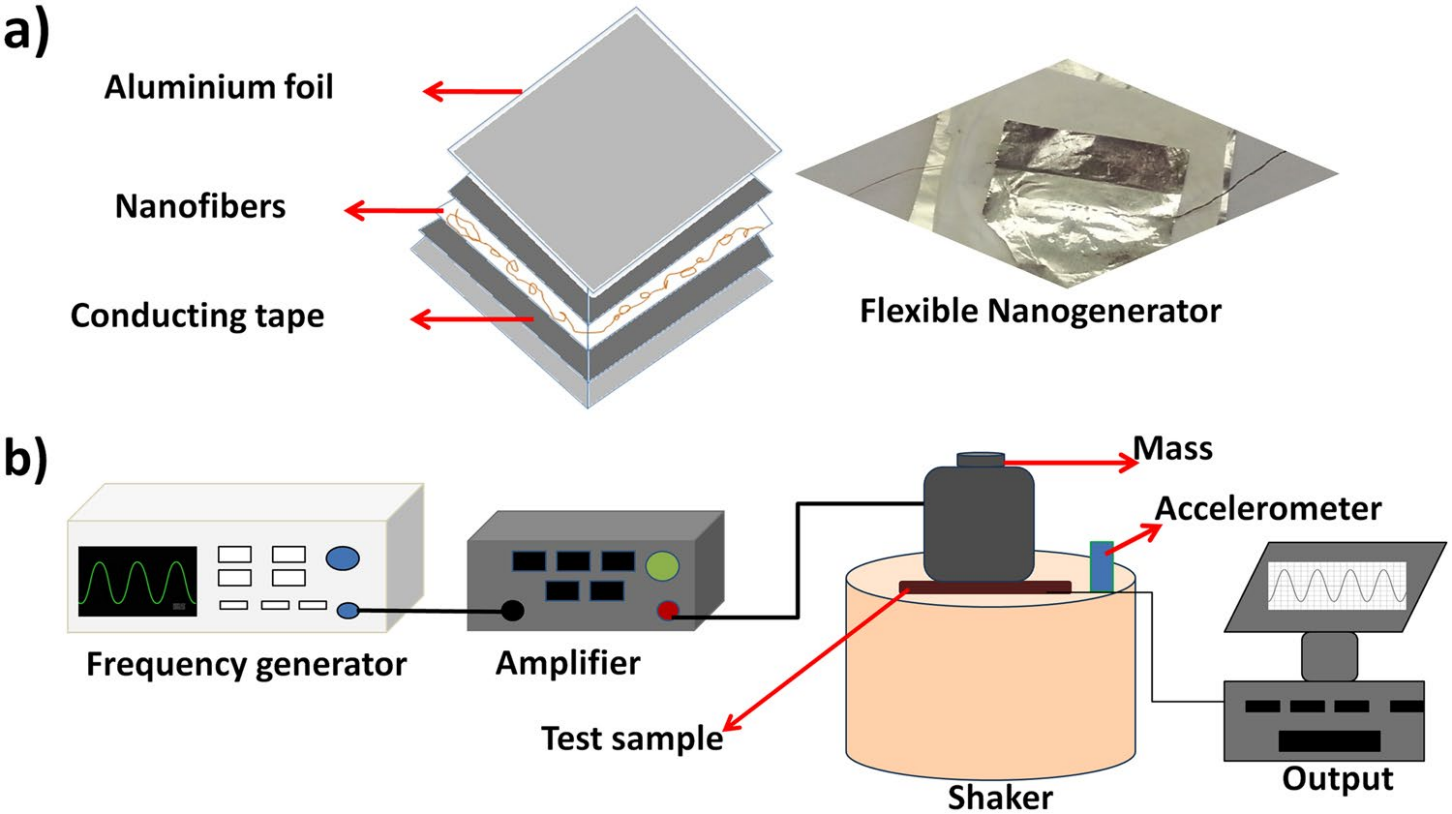
Schematic representation of (**a**) flexible nanogenerator and (**b**) piezoelectric experimental setup.

The piezoelectric property of the samples were analysed by means of an experimental setup consisting of a frequency generator, shaker and the output measurement system. A schematic of the PVDF-HFP composite nanofibers based energy harvester is shown in Fig. 2 along with the experimental setup. The nanogenerator shown in Fig. 2a was made by silver electroding at both surfaces and wires were attached through aluminium foil electrodes. Conducting carbon tape was used to fix the device to the substrate. The sample was placed on the top of a shaker and specific weight was placed over it. An amplified sinusoidal voltage generated by a frequency generator was sent to the vibrating shaker, which vibrates according to the weight placed on the top of it (Fig. 2b). The output voltage generated by the sample vibration was measured by a signal processor connected to the computer and used for evaluating the piezoelectricity.

### Data availability

No datasets were generated or analysed during the current study.

## Results and Discussion

**Figure 3:**
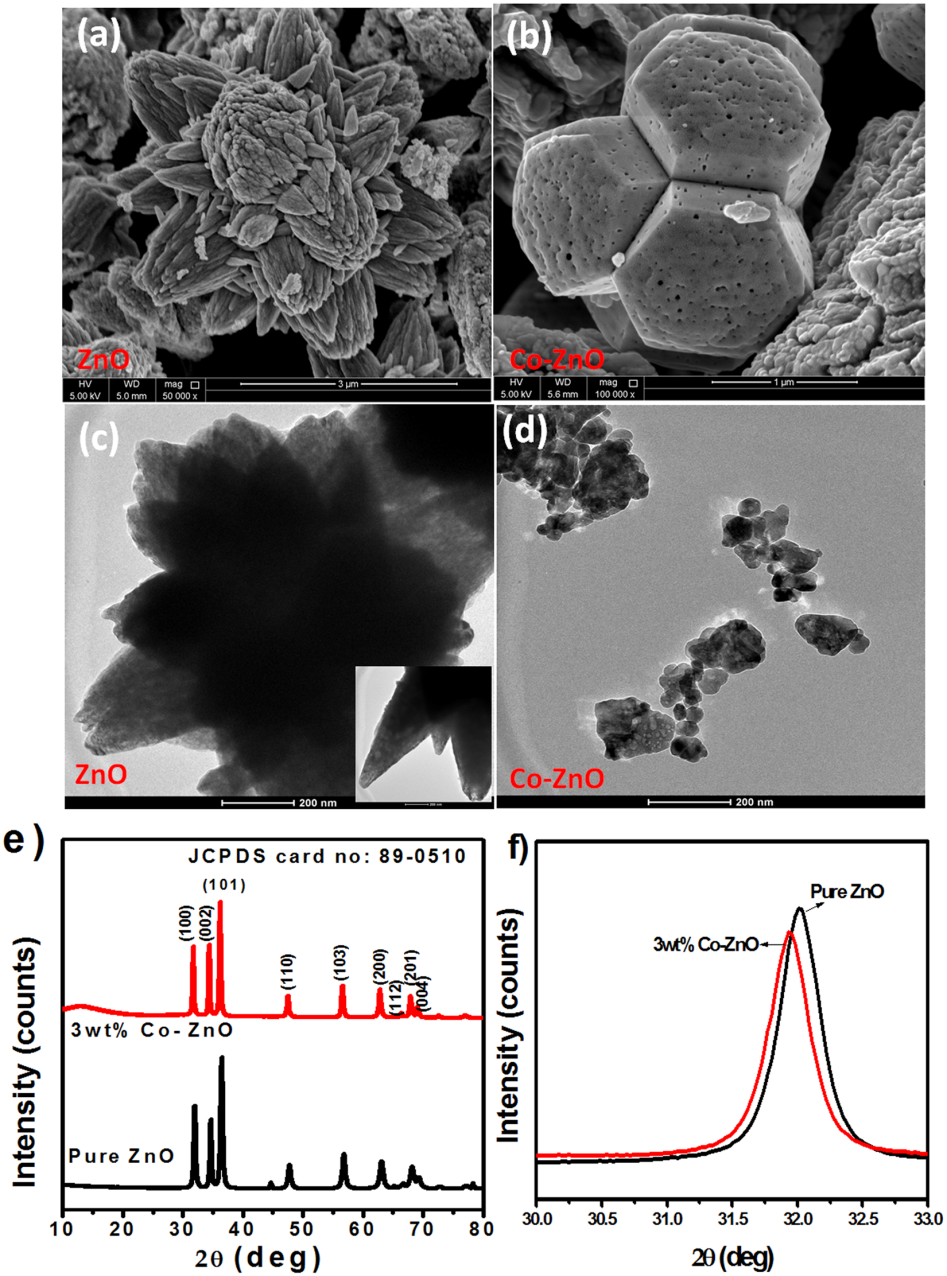
(**a**,**b**) SEM images (**c**,**d**) TEM images and (**e**,**f**) XRD patterns of undoped ZnO and Co-doped ZnO nanorods.

The surface morphology of undoped ZnO and Co-doped ZnO samples were characterized by SEM as shown in Fig. 3(a,b). For ZnO, the nanorods are oriented and assembled to form a flower like structure as given in Fig. 3a. Addition of the Co dopant, develops individual rods with hexagonal cross sections (Fig. 3b). The surface morphology was further confirmed by TEM studies (Fig. 3c,d), which also substantiates the flower like and hexagonal morphology for the undoped ZnO and Co-doped ZnO.

The structural properties of ZnO and Co-doped ZnO nanoparticles, synthesized by the hydrothermal method were characterized by X-ray diffraction technique and the obtained patterns are shown in Fig. 3e,f. All the diffraction peaks were well matched with the joint committee on powder diffraction standard (JCPDS card no: 89–0510) which indicates the hexagonal wurzite structure for the ZnO and modified ZnO. No diffraction peaks related to Co agglomeration or cobalt oxide can be observed, which implies that the crystal structure of ZnO does not change due to the incorporation of Co ions. This also suggests the uniformly substituted Co ions in the ZnO lattice sites.

Figure 3f shows the effect of Co doping in ZnO lattice, it is observed that the (100) peak is shifted towards a lower 2θ value when cobalt is incorporated in ZnO and also the intensity of the peak is decreased and broadened for Co-doped ZnO sample, confirming the decreased crystallite size. The crystallite size is an important parameter for the enhancement of output performance of the nanogenerator^16^. According to Paria *et al*.^17^, the piezoelectric response was increased with a decrease in the size of the nanoparticles. We have investigated the crystallite size of undoped and Co-doped ZnO nanoparticles following the De-bye Scherrer’s formula^18^.

1 $$D=\frac{0.9\lambda }{\beta \,cos\theta \,}$$

Where λ is the X-ray wavelength (1.5406 Å), β is the full width at half maximum and θ is the Braggs angle. The calculated crystallite size for undoped ZnO and Co-doped ZnO were 23 nm and 18 nm respectively, confirming the decreased crystallite size with the addition of cobalt ions.

**Figure 4:**
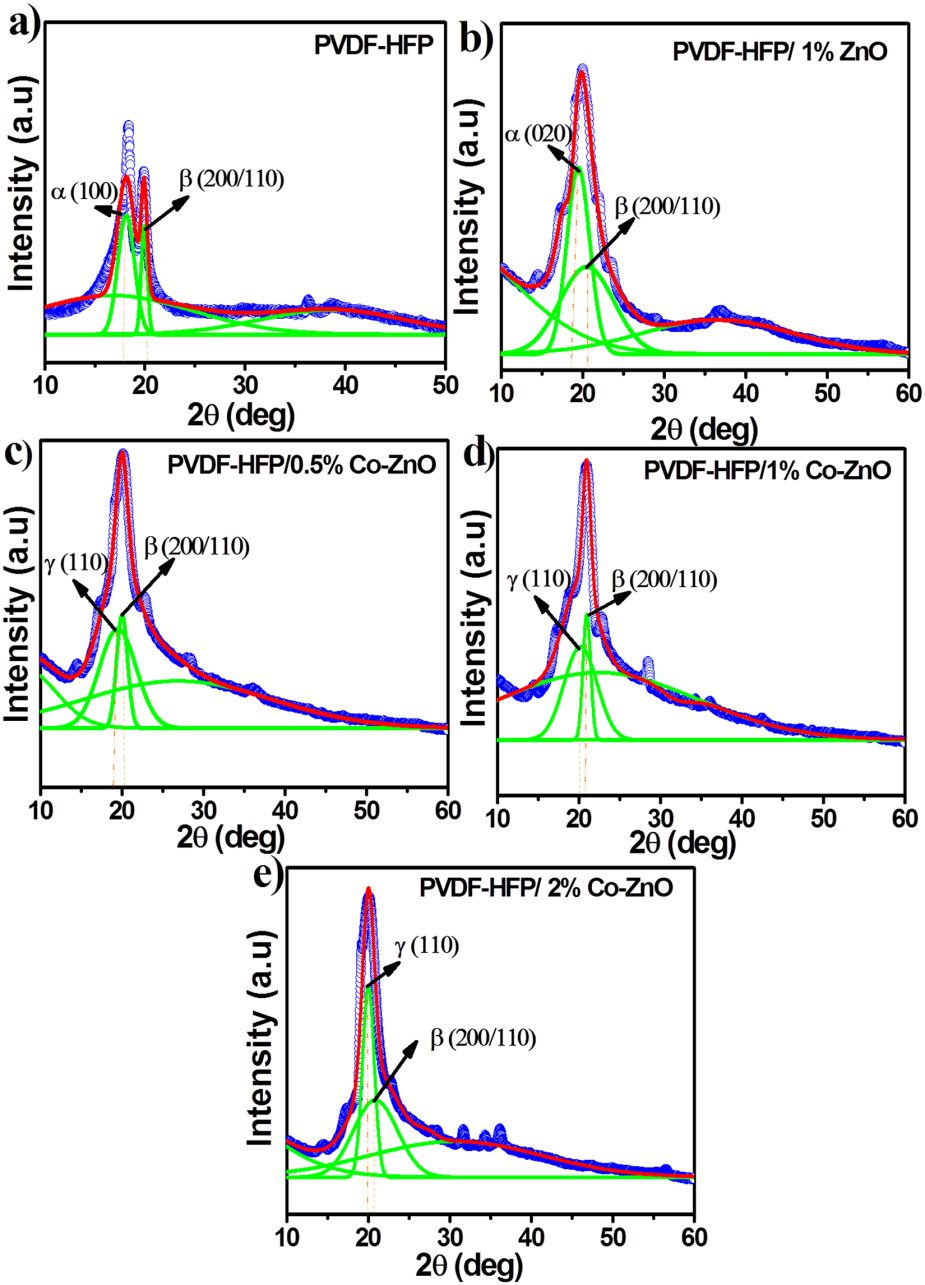
x-ray diffraction patterns of neat PVDF-HFP and its nanocomposites.

The phase and crystallinity of the neat PVDF-HFP and PVDF-HFP/Co-ZnO nanofibers were studied by analysing the X-ray diffraction pattern as shown in Fig. 4. The observed diffraction peaks correspond to the semicrystalline PVDF^19–21^. The crystalline α, β and γ phases of PVDF-HFP were well- fitted with a Gaussian function for all the samples and the electroactive polarized α, β and γ phases were calculated from the de convoluted peaks.

The deconvoluted peaks observed at 17.8° (100) and 18.6° (020) correspond to α- crystalline phase of PVDF-HFP whereas the peaks observed at 19° and 20.1° correspond to the γ- crystalline phase. The peak at 20.6° (200/110) corresponds to the β- crystalline phase. With ZnO loading (1 wt.%) the deconvoluted peaks were shifted towards higher 2θ value of 18.6° compared with the 17.8° α–phase peak of neat PVDF-HFP. As can be seen from the Co-doped ZnO filled systems (0.5 wt.%, 1 wt.% and 2 wt.%), the α- peaks were gradually disappeared and new peaks appear at 19° and 20.1° which clearly indicates the formation of a metastable γ-phase^22–25^.

It is also observed from Fig. 4 that the increase in Co-doped ZnO nanofiller concentration enhanced the β-phase for PVDF-HFP/Co-ZnO. A similar result was reported by Kumar *et al*.^26^, with doped AlO-rGO filled PVDF-HFP composite system, in which the β-phase crystallinity was enhanced. In addition, the diffraction peaks correspond to Co-doped ZnO nanoparticles were also observed in the range of 30°–60° for the PVDF-HFP/Co-ZnO nanocomposites. It is concluded from the XRD results that the addition of the Co-doped ZnO nanofiller enhanced the β-phase formation in the PVDF-HFP nanofibers. The intensity of the β-phase peaks increases with the increasing the Co-ZnO filler concentration as well (Fig. S1 in supporting information).

**Figure 5:**
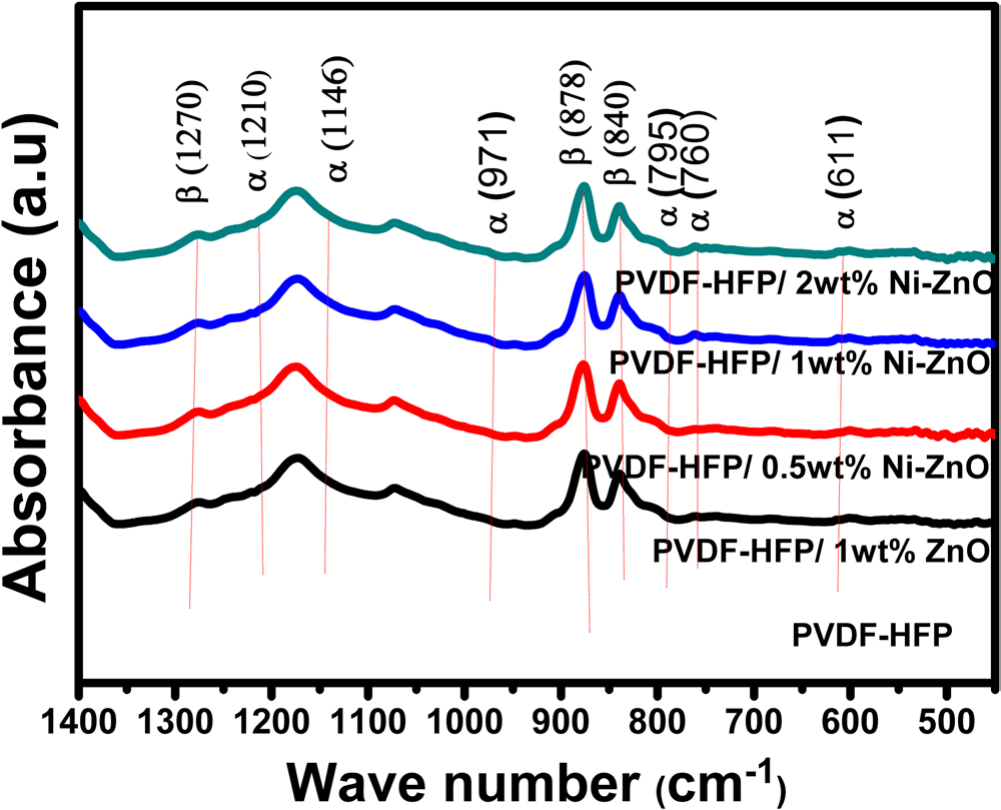
(**a**) FTIR spectra of neat PVDF-HFP and the nanocomposites.

The crystallinity of PVDF-HFP/Co-ZnO nanofibers were further analyzed by FTIR spectra and represented in Fig. 5. The vibrational peaks observed at 611, 760, 795, 1146, 1210 cm^−1^ were due to the α-phase of PVDF-HFP^27–29^. The peaks at 1270, 840, 878 cm^−1^ correspond to the β-phase^30^. It is clear from the FTIR spectra that the intensity of the peak at 840 cm^−1^ for the doped samples is higher than the neat PVDF-HFP nanofibers. It can also be observed that the α- phase at 611, 760, 795, 971, 1146 and 1210 cm^−1^ disappear in Co-ZnO nanocomposites confirming the increase in β-phase with the filler concentration. Many researchers have reported that the surface charges on the Co-doped ZnO nanofillers interact with the molecular dipoles (CH_2_ or CF_2_) of PVDF-HFP and improves the β- phase content of the composites^31,32^. This can also be explained on the basis of positive charges that present in the Co-ZnO nanofillers that interact with –CF_2_- dipoles of the PVDF-HFP segments in the nanocomposites. The peaks at 3030–2930 cm^−1^ wavelength (Fig. S2, Supporting Information) are assigned to the symmetric (ν_s_) and asymmetric (ν_as_) stretching vibration bands of PVDF-HFP and its nanocomposites. The symmetric and asymmetric vibration bands shift towards lower frequency region in the composites when compared to the neat polymer. This indicates the interaction of surface charges of the Co-doped ZnO nanoparitlces with CH_2_ and CF_2_ dipoles of PVDF-HFP matrix^33^. In addition, the spectra exhibited a broad band in the 3800–3200 cm^−1^ region (Fig. S2, Supporting Information) due to the formation of intermolecular H-bonds between the PVDF-HFP chains or between the filler and polymer in the nanocomposite. This H- bond is responsible for the alignment of different dipoles of PVDF-HFP^34^.

The crystalline β-phase proportions within the electrospun PVDF-HFP fibers are calculated by the following formula^35^.

2 $$F(\beta )=\frac{{A}_{\beta }}{1.26{A}_{\alpha }+{A}_{\beta }}$$

Where A_β_ and A_α_ are the area of absorption bands at 840 and 761 cm^−1^. The calculated values are shown in Table 1, from which it is clear that, compared to the neat PVDF-HFP, the β- crystalline phase content is more for the Co-doped ZnO composites.

**Table 1:** Melting and β**-** phase crystallinity data from DSC and FTIR spectra.

Samples	Degree of crystallinity (X_c_,%)	β-phase crystllinity
PVDF-HFP	21	37.5%
PVDF-HFP/1 wt% ZnO	23	42.7%
PVDF-HFP/0.5 wt%Co-ZnO	27	47.2%
PVDF-HFP/1 wt% Co-ZnO	32	52.5%
PVDF-HFP/2 wt% Co-ZnO	35	54.6%

**Figure 6:**
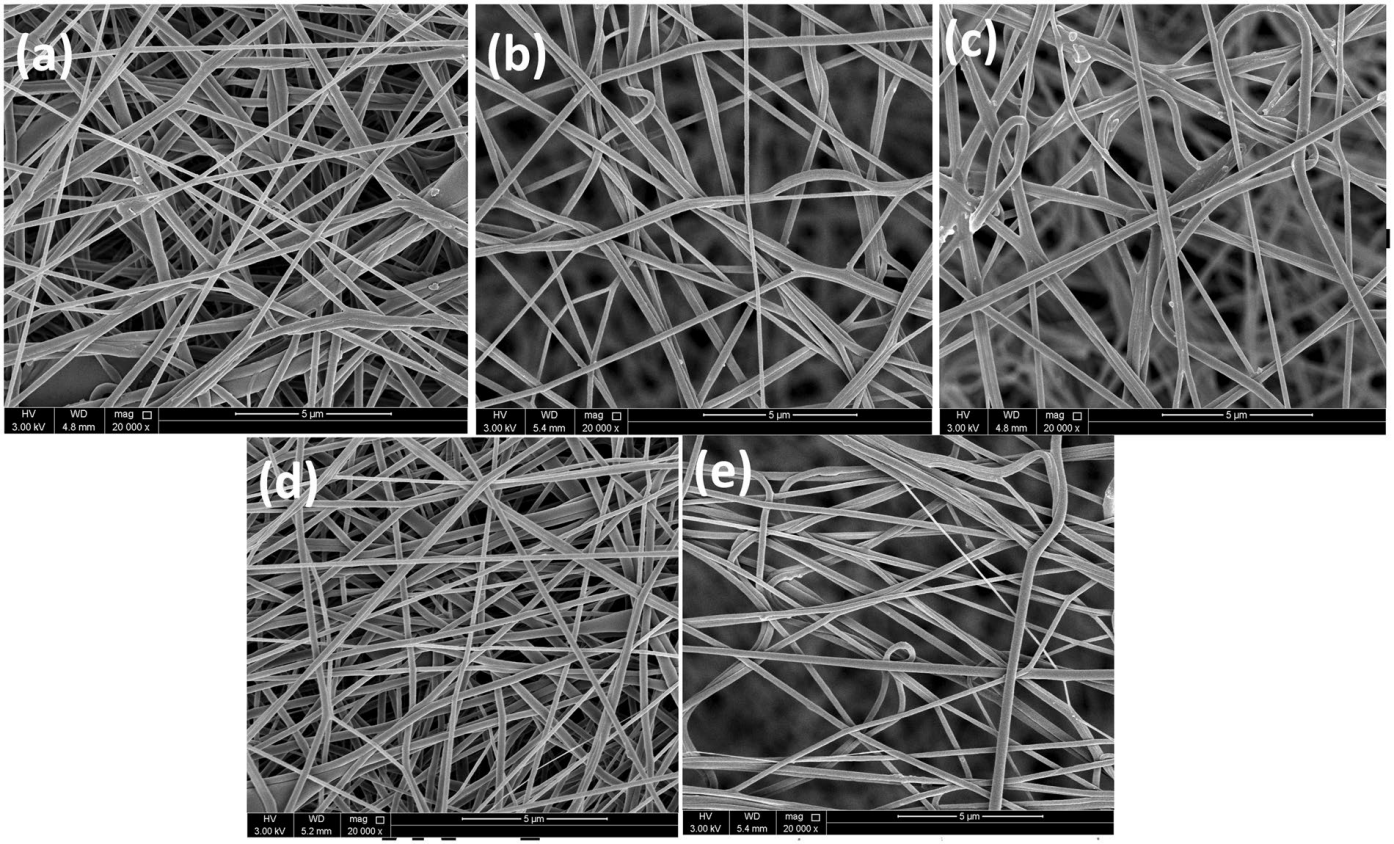
SEM images of (**a**) neat PVDF-HFP, (**b**) PVDF-HFP/1 wt.% ZnO, (**c**) PVDF- HFP/0.5 wt.% Co-ZnO, (**d**) PVDF-HFP/1 wt.% Co-ZnO, (**e**) PVDF-HFP/2 wt.% Co-ZnO.

The morphology of PVDF-HFP and PVDF-HFP/Co-ZnO samples were investigated by FE-SEM and the images are depicted in Fig. 6. The defect free electrospun fibers obtained for neat PVDF-HFP and the PVDF-HFP/Co-ZnO composites suggest no bead formation.

The formation of straighter, homogeneous, dense and defect free nanofibers is attributed to the increase in charge density of the polymer solution, as reported by Mandal *et al*.^33^. It is also seen that all the nanoparticles are distributed uniformly in the PVDF-HFP matrix with no agglomeration.

Differential scanning calorimetry (DSC) is used to study the thermal properties of the samples (Fig. S3, Supporting Information). The melting peaks were found to increase with increase in the filler loading, which can be due to the homogeneous dispersion of Co-doped ZnO nanofillers in the polymer matrix and also the nanofillers act as nucleating agents (that can increase the crystallinity of the composites)^36^. The degree of crystallinity (X_c_) was calculated using the following Equation^37^.

3 $${X}_{c}={\rm{\Delta }}{H}_{m100}/{\rm{\Delta }}{H}_{m}^{o}$$

Where ΔH_m_ and ${\rm{\Delta }}{H}_{m}^{o}$ are the melting enthalpies of the composites and neat polymer, respectively. The melting enthalpy for neat PVDF-HFP is fixed as 104.5 Jg^−1^. The calculated values for the crystallinity are also included in Table 1. This is in accordance with the FTIR results.

**Figure 7:**
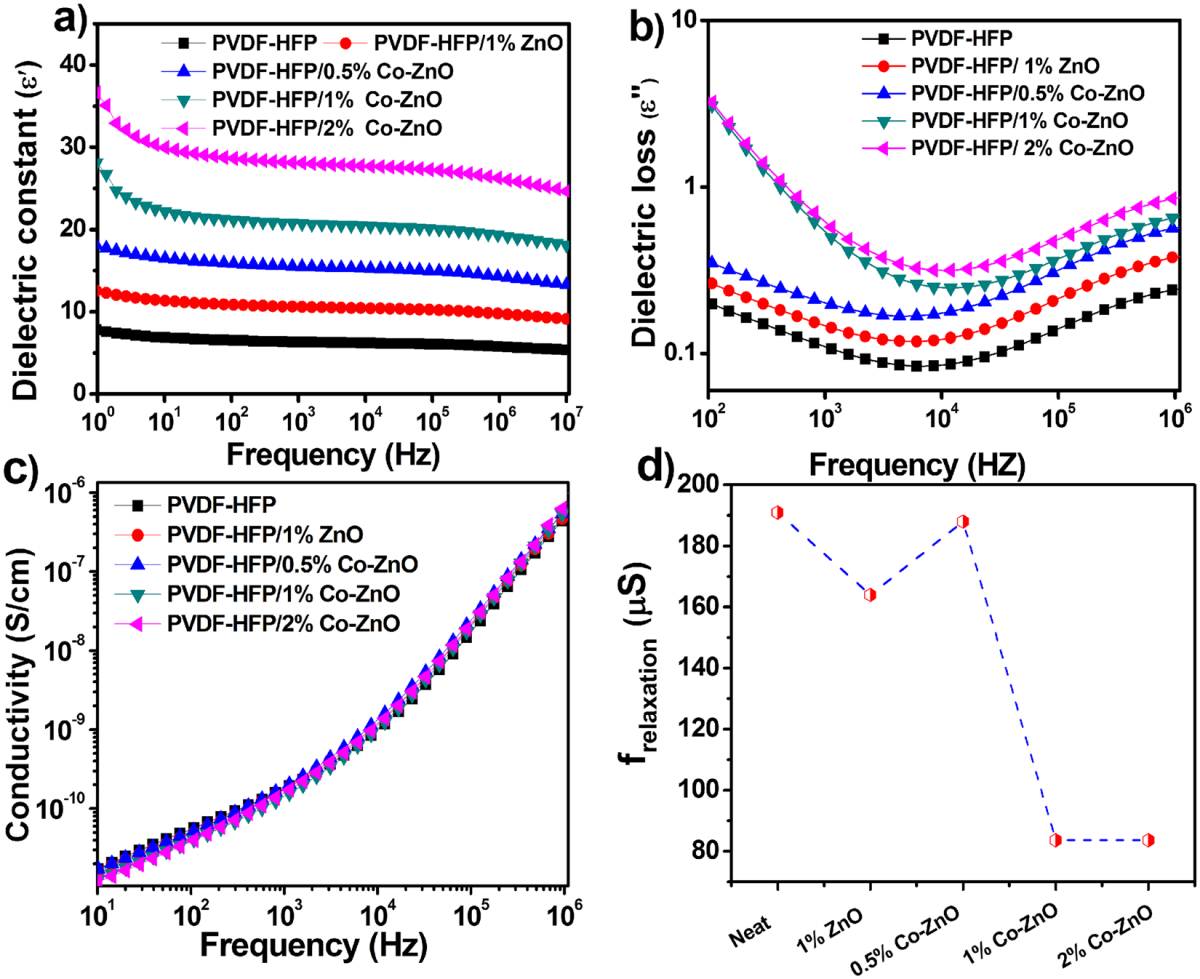
(**a**) Variation of dielectric constants (ϵ′) with frequency, (**b**) variation of dielectric loss (ϵ″) with frequency.

Figure 7a,b show the frequency dependent dielectric constant (ϵ′) and dielectric loss (ϵ″) of the neat PVDF-HFP and its nanocomposites. It can be seen that the ϵ′ increases by the addition of Co-ZnO nanofillers. The values for both ϵ′ and ϵ″ are high in the low frequency region due to space charge effects and interfacial polarization^38^. Also, the dielectric constant decreases at the high frequency region due to the slower dipole mobility^39^. The observed increase in dielectric constants with the filler loading is related with the decrease in filler-filler distances that enhances dipole-dipole polarization in the nanocomposites^40^. The high dielectric relaxations happening at low frequency called Maxwell-wagner relaxations are usually generated due to the interfacial effects arising between the fillers and polymer matrix^41^. For piezoelectric nanogenerator, high dipole polarization is important for the high output performance^42^. The conductive behaviour or electric heterogeneous nature of the composites or interfacial polarization between filler and polymer interfaces can be responsible for the high dielectric constant^43^. Figure 7c shows the variations in conductivity of the samples with respect to frequency. For all composites the conductivity shows similar behaviour with that of the neat polymer. This is because the lower concentrations of the ZnO and Co-ZnO semiconducting fillers within the composites. The reciprocal of the frequency at which the composite shows lowest dielectric loss is defined as the relaxation time and those calculated values are represented in Fig. 7d. The decrease in relaxation time from 191 μs for the neat polymer to 84 μs for the Co-doped ZnO sample confirms the dominating dipole polarization in the composite.

**Figure 8:**
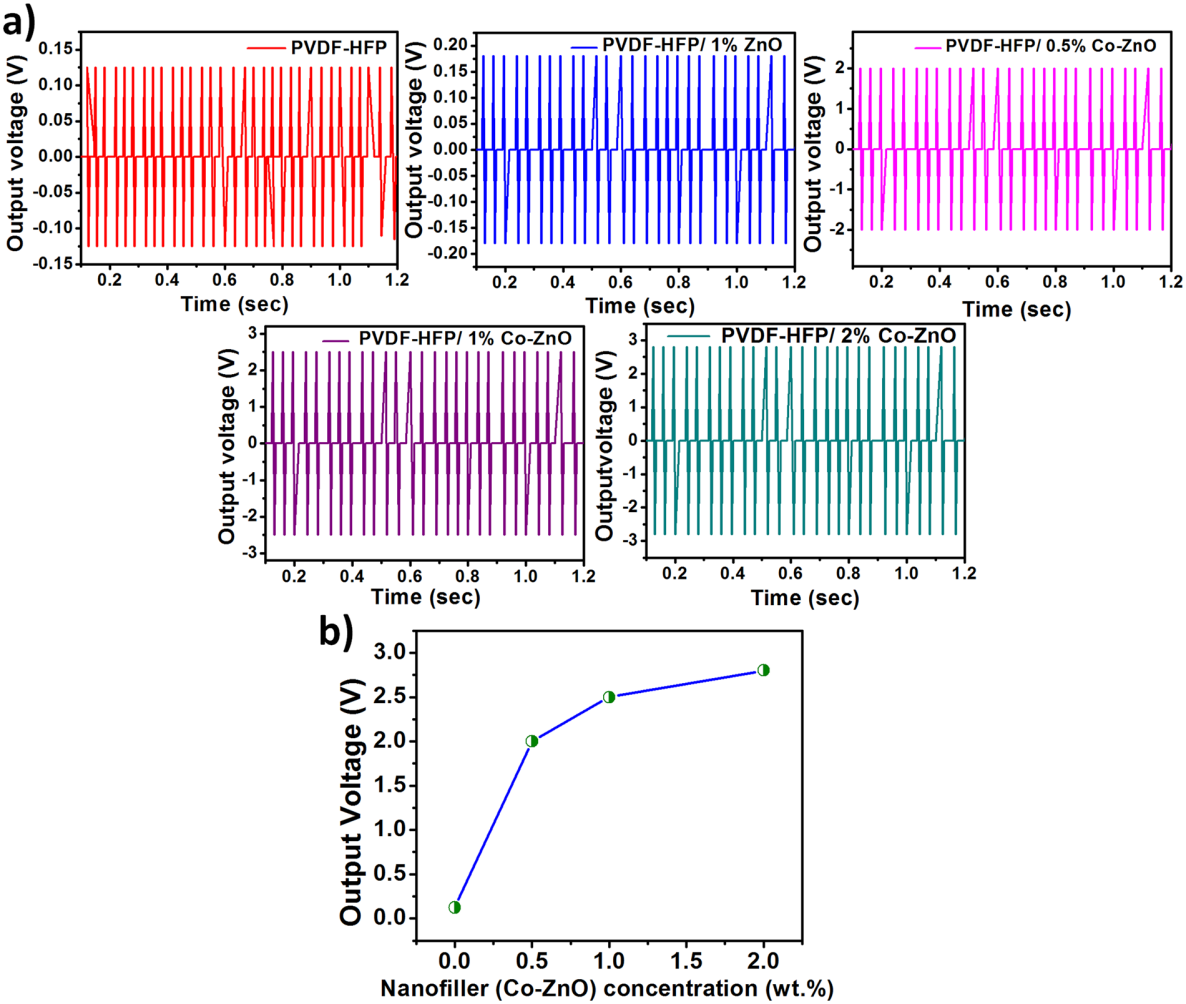
(**a**) Generation of output voltages from neat PVDF-HFP and its nanocomposites, (**b**) the output voltages as a function of the Co-doped ZnO filler loading.

Figure 8a shows the piezoelectric properties of PVDF-HFP and PVDF-HFP nanocomposites films. Piezoelectric output voltages of 2 V, 2.4 V and 2.8 V were respectively achieved from the nanogenerator containing filler loadings of 0.5, 1 and 2 wt.% of Co-ZnO. The output voltage was very low; 120 mV for neat PVDF-HFP and it is high for all the other nanocomposites. This can be attributed to the presence of piezoelectric ZnO in the nanocomposites in various concentrations^44,45^. In addition, the increase in output voltage is due to the relative proportions of polar β-phase present in the nanocomposites. It is evident from the XRD and FTIR results that the addition of Co-doped ZnO enhances the β-phase of PVDF-HFP. Thus, the piezopotential of the nanocomposites increases linearly with an increase in nanofiller concentration. This can be the reason for the enhanced output voltage for the Co-doped ZnO nanocomposites. Also, the XRD results confirmed the enhancement of β- crystalline phase in the Co-ZnO nanocomposites.

In other words, the β-phase for the polymer nanocomposite increases with increasing filler concentration and it can be attributed to the interaction between the oppositely charged Co-ZnO surface and the –CF_2_-/-CH_2_-dipoles of PVDF-HFP. This enhances the nanoparticles nucleation and the piezo electric polar β-phase can be stabilized by surface charge induced polarization^46^. In addition, by an application of mechanical force, the crystal structure of the PVDF-HFP/Co-ZnO fibers were deformed and the external pressure creates a potential in the Co-doped ZnO nanorods, which aligns the dipoles in the PVDF-HFP matrix^47^. These types of nanocomposites are suitable for touchable sensors such as those on the foot paths, bridges, shoes, vehicles and self-charging battery separators^48^. Fig. 8b shows the time-dependent generation of output voltage with the filler loading concentration under the constant tapping force of 2.5 N and the frequency at 50 Hz.

**Figure 9:**
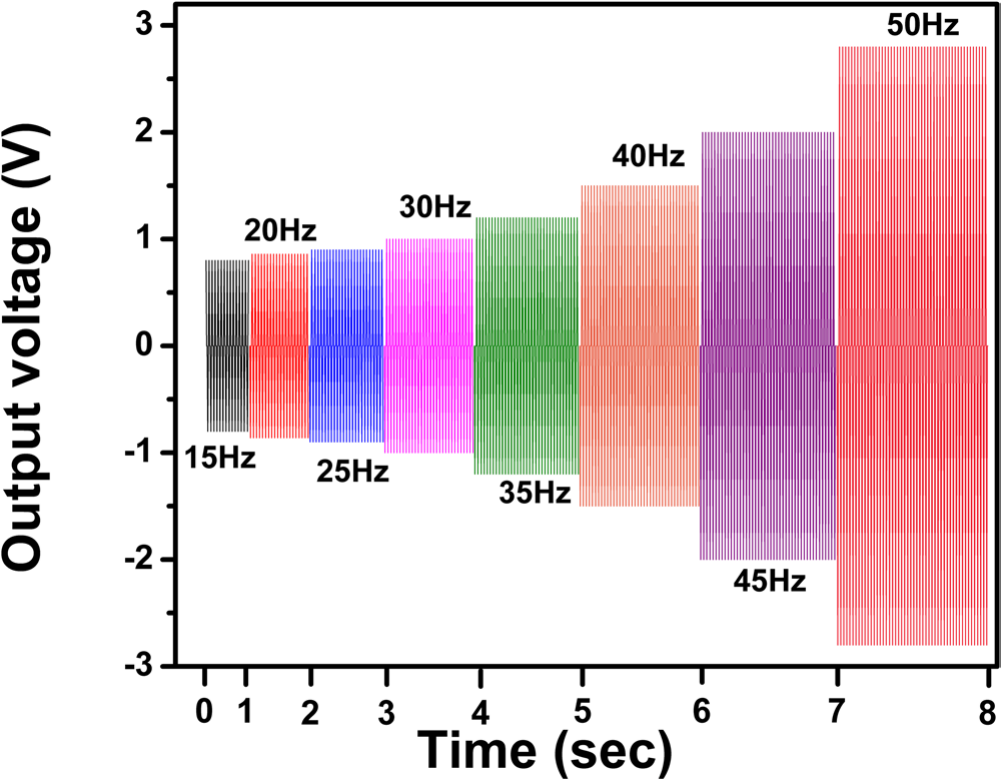
The output performance of the piezoelectric nanogenerator (PVDF-HFP/2 wt% Co-doped ZnO nanofibers) as a function of different frequencies.

Figure 9 shows the time-dependence of output voltage from the nanogenerator with 2 wt.% nanofiller loading over the full frequency range of mechanical vibration. It is important to study the relationship between the output performance of the piezoelectric nanogenerator and the different frequencies of applied force, because the mechanical energy from the ambient environment largely varies and is irregular^49^. In our case, the output voltage was measured repeatedly in the frequency range 15–50 Hz and from the figure, the output voltage slightly increases with the increase in frequency.

**Figure 10:**
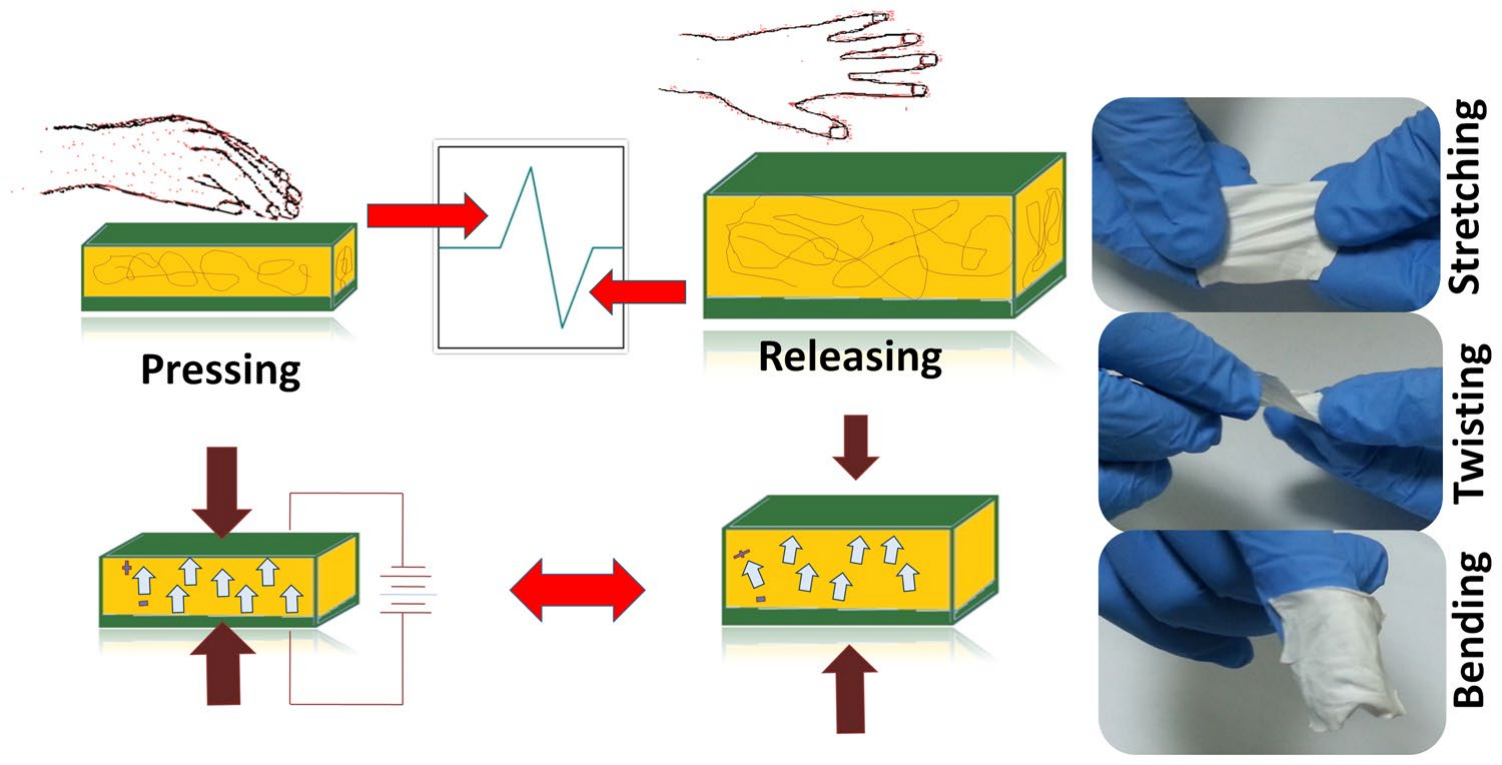
Working mechanism under pressing releasing mechanical force.

The power generation mechanism for the piezoelectric nanogenerator is shown in Fig. 10. It is established that, by an application of mechanical force, the electric dipoles in the crystal get oriented along a direction which is called the stress-induced poling effect^50^. When the force is released then the electron stream goes back through the external load, and hence both positive and negative voltage peaks can be observed under pressing and releasing during vibration. From the figure, a positive and negative piezoelectric potential was observed which is due to the deformation of the crystal structure. It can also be explained, in terms of the dipole alignment in the PVDF-HFP matrix and surface charges on the Co-ZnO nanorods. When the force is applied to a material, it creates a potential difference on the nanoparticles surface which aligns the dipoles uniformly in the direction of applied force. When the mechanical force is released, the electrons flow back to the electrode and produce electric signal in opposite direction^51^. The composite fibers upon stretching, twisting and bending modes are also represented in the figure, which supports the use of this material in self-powering devices of wearable electronics.

## Conclusion

In summary, we have successfully prepared PVDF-HFP/Co-ZnO nanofibers by electrospinning method. The structural characterization of the samples explored by XRD and FTIR studies shows higher β-phase content for PVDF-HFP/Co-ZnO (2 wt.%) nanofibers than the neat PVDF-HFP sample. The incorporation of Co-doped ZnO nanofillers enhances the nucleation and stabilization of the piezoelectric polar β-phase. It was inferred that the electrospinning method and Co-doped ZnO nanoparticles has a strong effect on structural and morphological properties of the nanocomposites which reveals a significant effect on piezoelectric properties. The highest piezoelectric output voltage of 2.8 V was observed for 2 wt.% Co-ZnO/PVDF-HFP nanofibers. The increasing output voltage is due to the impact of Co-doped ZnO nanofiller on the electroactive β-phase of PVDF-HFP in addition to the influence of modified ZnO. The enhanced piezoelectric efficiency suggests the use of these nanofibers in electronics and biomedical fields.

## Electronic supplementary material

Supporting Information
