# Author Correction: Stretchable Electrospun PVDF-HFP/Co-ZnO Nanofibers as Piezoelectric Nanogenerators

**DOI:** 10.1038/s41598-019-56281-6

**Published:** 2019-12-19

**Authors:** Hemalatha Parangusan, Deepalekshmi Ponnamma, Mariam Al Ali Al-Maadeed

**Affiliations:** 1 Center for Advanced Materials, Qatar University, P O Box 2713, Doha, Qatar; 2 Materials Science & Technology Program (MATS), College of Arts & Sciences, Qatar University, Doha, 2713 Qatar

Correction to: *Scientific Reports* 10.1038/s41598-017-19082-3, published online 15 January 2018

In Figure 5, Co-ZnO was incorrectly given as Ni-ZnO. In addition, the line on the graph for PVDF-HFP was omitted.

The correct Figure 5 appears below as Figure 1.

**Figure 1:**
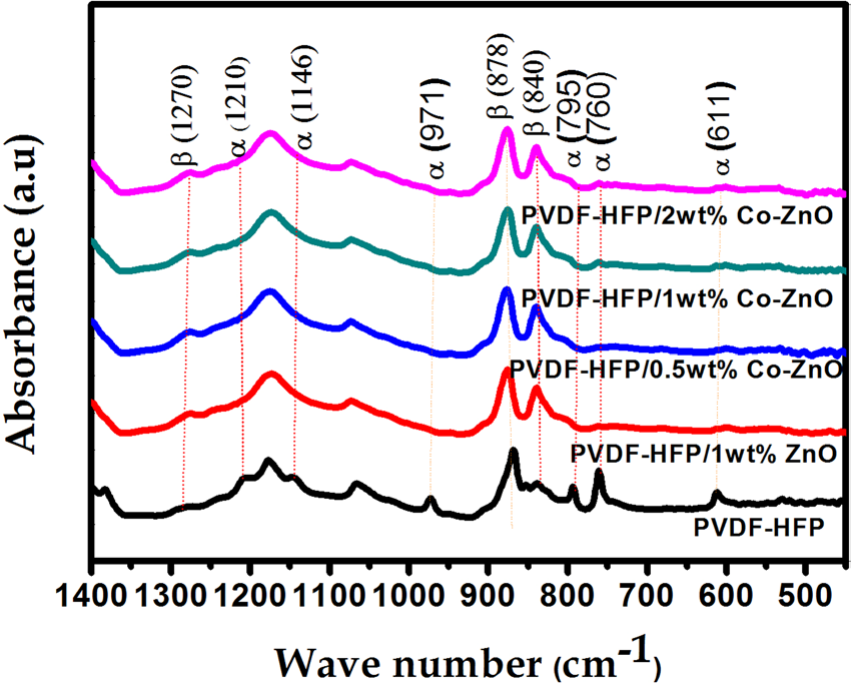
.

