# Supplementary material for: Stretchable Electrospun PVDF-HFP/Co-ZnO Nanofibers as Piezoelectric Nanogenerators
Source: Sci Rep. 2018 Jan 15;8:754. doi: 10.1038/s41598-017-19082-3 (PMC5768784; doi:10.1038/s41598-017-19082-3)
Supplement: Supplementary file 1 — Supporting Information [file 41598_2017_19082_MOESM1_ESM.pdf]

Supporting Information

Stretchable Electrospun PVDF-HFP/Co-ZnO Nanofibers as Piezoelectric Nanogenerators

Hemalatha parangusan[1], Deepalekshmi Ponnamma[1*], Mariam Al Ali Al-Maadeed[2]

[1]Center for Advanced Materials, Qatar University, P O Box 2713, Doha, Qatar

.[2]Materials Science & Technology Program (MATS), College of Arts & Sciences, Qatar University, Doha 2713, Qatar

Supporting Information S1: XRD spectra

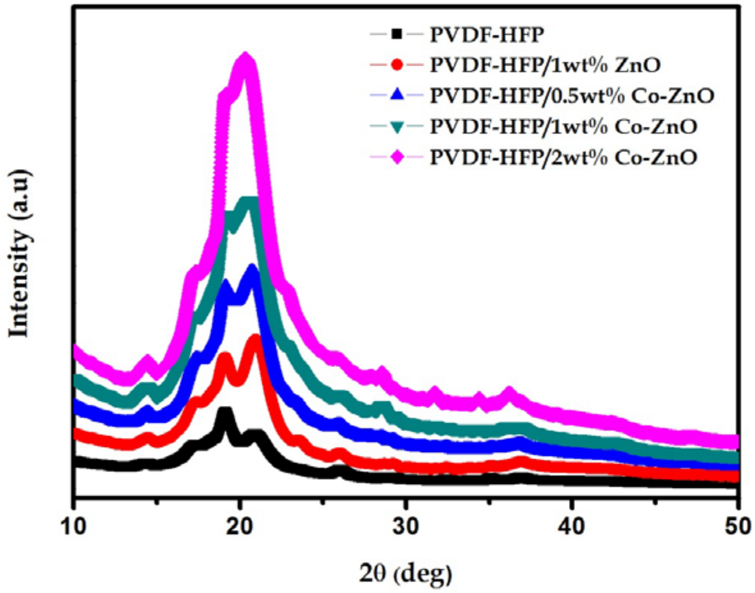

Fig. S1 X-ray diffraction pattern of pure PVDF-HFP and its nanocomposites

Supporting Information S2: FTIR spectra

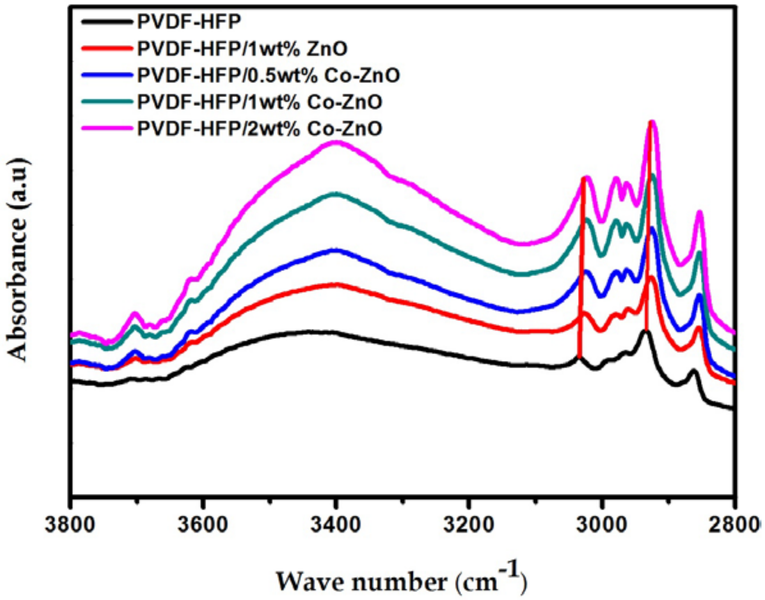

Fig. S2 FTIR spectra of pure PVDF-HFP and its nanocomposites

Supporting Information S3: DSC plots

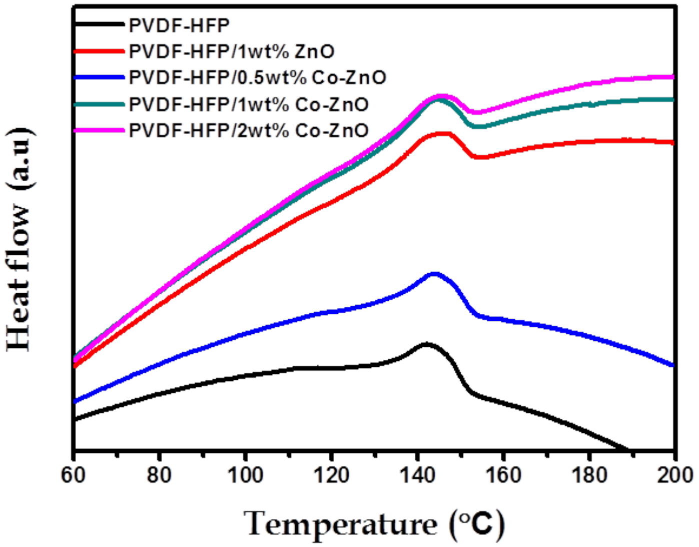

Fig. S3 DSC thermograms of pure PVDF-HFP and its nanocomposites.
